# Implementation of an Advance Care Planning Intervention in Nursing Homes: An International Multiple Case Study

**DOI:** 10.1093/geront/gnae007

**Published:** 2024-02-13

**Authors:** Kevin Brazil, Catherine Walshe, Julie Doherty, Andrew J E Harding, Nancy Preston, Laura Bavelaar, Nicola Cornally, Paola Di Giulio, Silvia Gonella, Irene Hartigan, Catherine Henderson, Sharon Kaasalainen, Martin Loucka, Tamara Sussman, Karolina Vlckova, Jenny T van der Steen, mySupport Study Group, Wilco Achterberg, Mandy Visser, Serena Fitzgerald, Danielle Just, Christine Brown Wilson, Gillian Carter, Laura Simionato, Catherine Buckley, Tony Foley, Siobhan Fox, Suzanne Timmons, Ronan O’Caoimh, Selena O’Connell, Catherine Sweeney, Emily Cousins, Kay De Vries, Josie Dixon, Karen Harrison Dening

**Affiliations:** School of Nursing and Midwifery, Queen’s University Belfast, Belfast, UK; International Observatory on End of Life Care, Lancaster University, Lancaster, UK; School of Nursing and Midwifery, Queen’s University Belfast, Belfast, UK; International Observatory on End of Life Care, Lancaster University, Lancaster, UK; International Observatory on End of Life Care, Lancaster University, Lancaster, UK; Medical Research Ethics Committees United, St. Andonius Hospital, Nieuwegein, The Netherlands; School of Nursing and Midwifery, University College Cork, Cork, Republic of Ireland; Department of Public Health and Pediatrics, University of Torino, Turin, Italy; Department of Public Health and Pediatrics, University of Torino, Turin, Italy; School of Nursing and Midwifery, University College Cork, Cork, Republic of Ireland; Care Policy and Evaluation Centre, The London School of Economics and Political Science, London, UK; School of Nursing, McMaster University, Hamilton, Ontario, Canada; Third Faculty of Medicine, Charles University, Prague, Czech Republic; School of Social Work, McGill University, Montreal, Quebec, Canada; Third Faculty of Medicine, Charles University, Prague, Czech Republic; Department of Primary and Community Care, Radboud University Medical Center, Radboudumc Alzheimer Center, Nijmegen, The Netherlands; Department of Public Health and Primary Care, Leiden University Medical Center, Leiden, The Netherlands; Department of Public Health and Primary Care, Leiden University Medical Center, Leiden, The Netherlands; School of Nursing and Midwifery, University College Cork, Cork, Ireland; School of Nursing, McMaster University, Ontario, Canada; School of Nursing and Midwifery, Queen’s University Belfast, Belfast, Northern Ireland, UK; School of Nursing and Midwifery, Queen’s University Belfast, Belfast, Northern Ireland, UK; Department of Clinical and Biological Sciences, University of Torino, Torino, Italy; Northridge House Education and Research Centre, St. Lukes Home, Cork, Ireland; Department of General Practice, University College Cork, Cork, Ireland; Centre for Gerontology and Rehabilitation, School of Medicine, University College Cork, Cork, Ireland; Centre for Gerontology and Rehabilitation, School of Medicine, University College Cork, Cork, Ireland; Centre for Gerontology and Rehabilitation, School of Medicine, University College Cork, Cork, Ireland; National Suicide Research Foundation, University College Cork, Cork, Ireland; School of Public Health, College of Medicine and Health, University College Cork, Cork, Ireland; Department of Medicine, University College Cork, Cork, Ireland; School of Nursing and Midwifery, De Montfort University, Leicester, UK; School of Nursing and Midwifery, De Montfort University, Leicester, UK; Care Policy and Evaluation Centre, London School of Economics and Political Science, London, UK; Dementia UK, London, UK

**Keywords:** Comfort care, Decision-making, Dementia, Family caregiver

## Abstract

**Background and Objectives:**

The inability of individuals in the advanced stage of dementia to communicate about preferences in care at the end-of-life poses a challenge for healthcare professionals and family carers. The proven effective Family Carer Decision Support intervention has been designed to inform family carers about end-of-life care options available to a person living with advanced dementia. The objectives of the mySupport study were to adapt the application of the intervention for use in different countries, assess impact on family satisfaction and decision-making, and identify costs and supportive conditions for the implementation of the intervention.

**Research Design and Methods:**

A multiple-case study design was chosen where the nursing home was the case. Nursing homes were enrolled from six countries: Canada, Czech Republic, Italy, Netherlands, Republic of Ireland, and United Kingdom.

**Results:**

Seventeen cases (nursing homes) participated, with a total of 296 interviews completed including family carers, nursing home staff, and health providers. Five themes relevant to the implementation of the intervention were identified: supportive relationships; committed staff; perceived value of the intervention; the influence of external factors on the nursing home; and resource impact of delivery.

**Discussion and Implications:**

There is a commonality of facilitators and barriers across countries when introducing practice innovation. A key learning point was the importance of implementation being accompanied by committed and supported nursing home leadership. The nursing home context is dynamic and multiple factors influence implementation at different points of time.

## Background and Objectives

Most people with dementia die in some form of residential care, which becomes an important place for their terminal or end-of-life care (Reyniers et al., 2015). In a recent international meta-analyses, it was estimated that 53% of residents in long-term care facilities were living with dementia. (Fagundes et al., 2021).Because people with advanced dementia struggle to communicate their care preferences, this poses a challenge for nursing home staff and family carers. In these situations, family carers often become important proxies to communicate care preferences (Jablonski et al., 2005; Robison et al., 2007). However, their role as a proxy decision-maker depends on them having accurate and timely information to facilitate such decisions. Family carers, however, can be disappointed by the lack of contact and meaningful communication regarding illness progression and feel unprepared to engage in care decisions (Hennings et al., 2010). Nursing home staff can also be reluctant to discuss end-of-life care due to a lack of understanding of the progression of dementia palliative and holistic care, and having the communication skills to conduct these discussions with residents and family carers. However, staff training can increase both competence and confidence in conducting advance care planning to help ensure that people receive medical care that is consistent with their values, goals and preferences during serious and chronic illness. For many people, this process may include choosing and preparing another trusted person or persons to make medical decisions in the event the person can no longer make his or her own decisions (Sudore et al., 2017).

One intervention designed to inform family carers on end-of-life care options for a person living with advanced dementia is the Family Carer Decision Support Intervention (Brazil et al., 2018). The effect of this intervention was demonstrated through a study that employed a cluster randomized control trial involving 24 care homes located in the United Kingdom. The primary outcome was family carer uncertainty in decision-making about the care of the resident (Decisional Conflict scale). There was evidence of a reduction in total Decisional Conflict scale score in the intervention group compared with the usual care group (−10.5, 95% confidence interval: −16.4 to −4.7; *p* < .001). There was also evidence that family carer satisfaction with care measured by the Family Perceptions of Care scale increased (8.6, 95% confidence interval: 2.3 to 14.8; *p* = .01). This cluster randomized trial indicated that it is feasible to implement the Family Carer Decision Support (FCDS) intervention in nursing homes with effective outcomes.

### The Family Carer Decision Support Intervention

The intervention consists of three components:

1. Training. A train-the-trainer model involves training nursing home staff to deliver the intervention (internal facilitators) who are supported with training and ongoing support by a trained facilitator external to the nursing homes (external facilitator). This approach involves e-learning and (digital) support resources to provide external facilitators with the skills required to train and support the internal facilitators.
2. Educational booklet and question prompt list. A Comfort Care Booklet was adapted to support the intervention (Arcand & Caron, 2005). The booklet is available in multiple languages, adapted to local contexts. This provides family carers with information so that they can better understand the risks and benefits of care options and the opportunity to actively participate in decision-making. It provides information on care for people with advanced dementia, decisions about the end of life, relief of symptoms, the final moments and after the death. The booklet has shown evidence of high levels of acceptability among family carers and healthcare providers and is identified as a best practice instrument (van der Steen et al., 2011). This is supported by a question prompt list, used by family carers as discussion prompts (Bavelaar et al., 2022).
3. Family care conference. After the provision of the “Comfort Care Booklet,” a structured conference is arranged involving a trained nursing home staff person (internal facilitator), family carer(s), and significant others as identified by family carer(s). The structure of the 1-hr conference (preparing, conducting, documentation, and follow-up) is based on clinical practice guidelines developed for conducting family meetings, underpinned by the REMAP framework (Childers et al., 2017). In the conference, the internal facilitator takes a personalized approach to review, discusses the question prompt list and the contents of the booklet with family participant(s), facilitating awareness of and a discussion about comfort care practices and preferences at the end of life.

Given the known impact of the Family Carer Decision Support Intervention in the context of the United Kingdom, it is important to understand whether this resource can be implemented and “scaled up” across different country contexts. The objectives of the mySupport study were fourfold:

(1) Adapt the application of the Family Carer Decision Support Intervention for use in different countries;
(2) Assess the impact of the intervention on family satisfaction with care and decision-making on goals of care;
(3) Identify supportive conditions for the successful implementation of the intervention; and
(4) Identify the costs associated with implementing the intervention.

A recent publication by the research team addressed the second objective of the study, reporting that family caregivers who participated in the intervention reported less decision-making uncertainty and more positive perceptions of care after the intervention. Further, the number of advance decisions to refuse treatment was significantly higher after the intervention. However, the number of other advance decisions or hospitalizations was unchanged (Bavelaar et al., 2023). In this article, we report on the latter two objectives that identify the supportive conditions for the implementation of the Family Carer Decision Support Intervention and the cost of resources associated with implementing the intervention.

## Research Design and Methods

### Design

A multiple-case study design was chosen (Yin, 2018). A case study approach enables understanding of the intervention implementation process and identification of factors, which determine how well the intervention may work in different contexts (Walshe, 2011). The study protocol is published (Harding et al., 2022).

### Definition of the Case

Nursing homes were implementing the Family Carer Decision Support Intervention. Nursing home refers to an institutional setting in which care is provided on-site 24 hr a day, including on-site nurses and attending medical staff (Sanford et al., 2015). Nursing homes were considered if they were responsible for the care of people living with advanced dementia.

### Initial Theoretical Propositions

Initial theoretical propositions were developed to guide the case study (Yin, 2018). These were initially developed using existing empirical evidence, as outlined in Table 1.

**Table 1.** Initial Theoretical Propositions

Theme	Theoretical propositions
Characteristics of the Family Carer Decision Support intervention (FCDS)	The FCDS intervention is viewed as easy to use by nursing home staff. Nursing home staff and family carers can see the positive effects resulting from its use. The FCDS intervention can be adapted to individual nursing home needs or practice.
External pressures on the nursing home	Implementation of the FCDS intervention within a nursing home meets external (regulatory) requirements or guidelines.
Characteristics of the nursing home environment	The leadership in the nursing home is committed and involved in the implementation of the FCDS intervention. Nursing home staff responsible for delivering the FCDS intervention will be able to accommodate the responsibilities into their workload and the intervention is recognized as core to their work.

### Case Study Site Selection

A purposive approach to sampling nursing homes was used, taking account of features including geographical location, size of facility, and external academic links. Cases were invited to participate and selected across six participating countries: Canada, Czech Republic, Italy, Netherlands, Republic of Ireland, and the United Kingdom.

### Participants Within Each Case

All family carers who had a family member identified as having advanced dementia and not having decisional capacity to participate in advance care planning discussions were eligible to participate in the intervention. Nursing home managers, resident chart review, and consultation with family carers confirmed capacity status of the resident. Residents were assessed by the nursing home staff on the Functional Assessment Staging Test to measure cognitive impairment (Reisberg, 1988). External facilitators were identified by researchers in each locality. Eligibility for this role was that individuals had experience in training healthcare professionals in a nursing home setting. The nursing home manager in the participating nursing homes identified staff to be recruited to act as an internal facilitator. All participating homes had both trained internal facilitators and access to external facilitators (Table 2).

**Table 2.** Data Collection Schedule

Case data	Timepoint		
	Preintervention		Postintervention
	Enrollment	Phase 1	Phase 2
	−t1	t1	t2
Enrolment:			
Eligibility screen	X		
Informed consent	X		
Data collection:			
Home profile	X		
Environmental scan interviews with staff		X	
Environmental scan interviews with family carers		X	
Follow-up interviews with staff			X
Follow-up interviews with family carers			X
Interviews with external facilitators			X
Interviews with healthcare professionals			X
Use of online training by internal facilitator			X
Use of online training by external facilitator			X
Internal facilitator training received in person			X
Training delivered by external facilitator in person			X
Backfilling of internal facilitators shifts			X
Cost of materials			X
External facilitator timesheets			X
Internal facilitator timesheets			X

### Data Collection

We used the RE-AIM framework constructs (Reach, Effectiveness, Adoption, Implementation, and Maintenance) which are considered important for effective and sustainable implementation to guide the development of the data collection tools (Glasgow et al., 2019). Data collection occurred across two phases including first an environmental scan, and post-family care conference data collection. Data collection occurred between 2020 and 2021. The forms and timing of data collected are summarized in Table 2.

In the environmental scan prior to implementing the intervention, semistructured interviews were conducted with family carers, nursing aides, registered nurses (including internal facilitators), and nursing home managers. Interviews examined attitudes, level of support, barriers to implementation, and potential cooperation related to the intervention. Nursing home managers also completed a nursing home profile, which catalogued bed size, profit status, and presence of advance care planning policies. Interviews were conducted by country-level research staff who were master’s or doctoral educated. Research staff were trained collectively on data collection protocols. All research staff participated in dementia palliative care training program, which included a mentorship scheme with senior investigators, knowledge exchange activities among researchers, speaker symposiums, as well as training in presenting and writing for publication. Staff-level researchers were responsible for data collection, and trained external facilitators were charged with the responsibility of supporting the facilitation of the intervention.

Phase two data collection took place approximately 6–8 weeks after the family care conference. Semistructured interviews were conducted with external facilitators, internal facilitators, nursing home managers, healthcare professionals, and family carers to explore conditions that may influence the implementation of the intervention. Topic themes explored the perceived usefulness of the intervention and integration in the resident care plan and the impact of the intervention on the work experience of the nursing home staff. Furthermore, interviews explored resource impacts that implementation had in the nursing homes. Family carer acceptability of the intervention was also assessed. Interviews were conducted in the local language. In addition, data were collected to enable an exploration of the economic aspects of the implementation. This included data on the direct and indirect costs of training, such as training modes (face-to-face in person or virtually, online training platform), backfilling of facilitator shifts, wage costs of facilitators, costs of materials (e.g., electronic tablets, printing of manuals, and comfort care booklets).

### Data Analysis

Data were analyzed within each nursing home case, followed by cross-case analysis within and then across countries. Analysis was driven by interrogation of the initial theoretical propositions, matching an observed pattern across cases with an expected pattern (theoretical proposition) and deciding whether these patterns match (resulting in a confirmation of the proposition) or do not match (resulting in a disconfirmation of the theoretical proposition; Yin, 2018, p. 168). Patterns and generalizations across cases were identified, which resulted in the development of themes that generated final theoretical propositions for implementing the intervention in nursing homes.

Interviews were digitally recorded and transcribed verbatim in the native language of each case. Codebooks and individual case nursing home templates were developed in an iterative process with researchers from each partner country. Researchers from partner countries participated in regular online sessions to ensure a standardized coding process. Codebooks were used in conjunction with the nursing home profile to develop an individual case template. The codebooks and the individual nursing home case templates informed the joint development of a cross-case template, which was populated with findings from each case. Cases developed at the country level were translated into English by country-level researchers to enable cross-country analyses. A framework analysis was applied to the cross-country analysis by three experienced qualitative researchers located in the United Kingdom. Framework analysis (Ritchie et al., 2013) enabled a systematic description of all aspects of an implementation process and identified relevant facilitating and hindering factors.

### Economic Analyses

Indicative costs of the train-the-trainer intervention were calculated in 2021 prices. Costs of non-UK sites were converted to GBP. Total costs were aggregated across all countries, and unit costs (such as the cost of training per internal facilitator hour) calculated by dividing these costs by the sum of the units of interest (e.g., hours) across countries. Qualitative interviews included prompts to explore resource impacts that implementation of the FCDS intervention had in the nursing homes (e.g., time spent by facilitators and other staff, costs to providers).

### Public Engagement

The mySupport study established an international Strategic Guiding Council including family carers of persons with dementia from all six countries. A local Public Advisory Group was also established by most of the countries. Members in these groups supported local adaptation to the educational intervention and provided input to the research process.

### Ethics Approval and Participant Consent

All project participants provided informed consent. The study and consent processes were reviewed by the relevant ethics review boards in each partner country.

## Results

Thirty-eight nursing homes were invited to participate in the project across the six countries. Twenty-one nursing homes declined for multiple reasons including staff capacity, responding to a coronavirus disease 2019 (COVID-19) outbreak, or simply not responding to the invitation. Seventeen nursing homes were recruited for intervention delivery across the six countries and received training, of which 13 went on to implement the intervention, delivering family care conferences with recruited family carers. Details of cases are in Table 3.

**Table 3.** Overview of Cases That Implemented the Intervention

Variables	Canada		Netherlands		Republic of Ireland		Czech Republic		Italy		United Kingdom		
*NH Characteristics*	NH1	NH2	NH1	NH2	NH1	NH2	NH1	NH2	NH1	NH2	NH1	NH2	NH3
Ownership^a^	1	1	2	2	1	2	2	2	1	2	2	2	2
Profit status^b^	1	1	1	1	1	2	2	1	1	1	2	2	2
Advance care planning policy	Yes	Yes	Yes	Yes	Yes	Yes	No	No	No	No	Yes	Yes	Yes
Size (no. of beds)	387	221	105	165	128	68	116	101	106	80	75	73	40
*Participant Characteristics*													
Family carers													
No. who participated in the intervention	13	10	3	13	6	5	7	17	11	2	7	6	1
Relationship to resident													
Spouse	1	0	0	4	0	1	1	2	0	0	0	3	1
Child	12	8	3	8	6	3	4	12	9	2	7	3	0
Extended family	0	0	0	1	0	0	1	1	2	0	0	0	0
Friend	0	0	0	0	0	0	1	1	0	0	0	0	0
Other	0	2	0	0	0	0	0	0	0	0	0	0	0
Internal facilitators													
No. Trained	3	1	5	5	4	3	2	4	1	2	3	3	3
External facilitators													
No. of facilitators	1	1	2	0	1		1		1	1	1	1	2

*Notes*: NH = nursing home.

^a^Ownership: 1 = independently owned nursing home, 2 = nursing home run as part of a chain.

^b^Profit status: 1 = nursing home run for nonprofit, 2 = nursing home run for profit.

Reasons for the recruited nursing homes not completing the delivery of the intervention (3 = United Kingdom, 1 = Czech Republic) included COVID-19-related factors such as dealing with a COVID-19 outbreak during the course of the study, or managing staff fatigue subsequent to an outbreak. Other factors attributed to nonimplementation included addressing competing practice/training, for example, infection control, vaccination program, or administrative priorities within the implementation period.

The predominant professional training of the internal facilitator was nursing; however, three internal facilitators had a social work background. External facilitators who were responsible for supporting internal facilitators largely held a nursing background. External facilitators held affiliations in a range of institutions including hospice, university, and hospital settings. The number of family carers who participated in the intervention varied, ranging from 1 to 17 across nursing homes.

### Conditions That Influence the Implementation of the Intervention in Nursing Homes

The total number of completed interviews was 296. During the environmental scan, 136 interviews were completed, and following the delivery of the intervention, 160 interviews were conducted. Family carers completed 134 interviews, nursing home managers 34, internal facilitators 77, external facilitators 11, nursing home staff 28, and health professionals 12. Cross-case analysis identified five themes relevant to implementation of the intervention in nursing homes and final theoretical propositions (Table 4)

**Table 4.** Final Theoretical Propositions

Theme	Theoretical Propositions
Trust and supportive relationships.	Trusting and supportive relationships between all stakeholders are key factors in successful implementation of the intervention. Implementation of the FCDS intervention addresses family priorities.
Committed staff and leadership toward improving practice.	The FCDS intervention requires committed and engaged leadership from individual(s) in the nursing home for implementation to be successful. Nursing homes culture embodies readiness and openness to change, as highlighted by positive attitudes and recognizing the intervention as core to their work.
The perceived value of building skills in communication with families.	Nursing home staff responsible for delivering the intervention feel capable and comfortable engaging families in EOL discussions; this is established via accessible and impactful training and support. The success of the intervention depends on adapting the FCDS intervention to local contexts and supporting implementation.
Factors external to the nursing home.	Implementation of the FCDS intervention within a nursing home was influenced by professional guidelines or public health policies on COVID-19 and infection control.
Resource impact on delivering the intervention.	Nursing home staff responsible for delivering the FCDS intervention can accommodate the responsibilities into their workload.

*Note*: FCDS = Family Carer Decision Support Intervention.

### Theme 1. Trust and Supportive Relationships

Trust was viewed by nursing home staff and family carers as essential to their relationships in effectively delivering the intervention both in recruiting participation and facilitation in the family care conferences. Family carer perception of the personal resources of staff including empathy, knowledge, and skill facilitated trust among family carers. Embedded in the trusting relationship was confidence by family carers that nursing home staff would take care of their family member residing in the home, acknowledging vulnerability and dependency:

I felt when I was there that I would put my trust in the nurse who was talking to me, to look after mum, you know I really did. (UK—Northern Ireland, family carer)

Trust was viewed as dynamic, to be earned, when family members developed confidence in the abilities of nursing home staff and felt that the staff had the best interests of their family member:

I know they don’t know me but they trusted me at the end. Especially when I get back to them—I see the appreciation. (Canada, internal facilitator)

I have to say that if I didn’t know the relatives very well it might have been a bit uncomfortable in the beginning you know, because it’s such a sensitive subject, I think for their sake if they trust you and know you it’s much easier for them to discuss this with someone that they know personally care after the relative. (UK—Northern Ireland, internal facilitator)

When relationships between families and staff were new and trust was not yet developed facilitating family carers to participate in the intervention was difficult:

Almost all family carers are new and we haven’t had a chance to build trust and cooperation with them. (Italy, internal facilitator)

A key factor in successful implementation of the intervention was the perception by nursing home staff and family members that the intervention addressed family priorities. Family members frequently reported that pain management and spiritual care were their main care priorities:

… before the actual beginning of these conferences, they may not have admitted at all or simply could not talk about this topic and actually based on the conferences they were so I think a lot of people were happy that the topic opened up. (Czech Republic, internal facilitator)

I found it very beneficial […] when the time comes she will be very well looked after, I know she’s not going to be in any pain, she will be as comfortable as possible. Because from the family conference it is all about comfort for someone at end of life. (Republic of Ireland, family carer)

The COVID-19 pandemic and accompanying infection control procedures did undermine trust between nursing home staff and families. Nursing home staff did report a hesitancy to talk about the end of life during the COVID-19 outbreak with family carers:

Many relatives resent us because they think we infected their loved ones [with COVID] and don’t want to talk to us. (Italy, internal facilitator)

### Theme 2. Committed Staff and Nursing Home Leadership to Improving Practice

Leadership committed to practice improvement in the nursing home was a key organizational factor toward successful implementation of the intervention, together with an organizational culture where staff saw themselves as supporting each other:

The whole team itself is very knowledgeable. Very collaborative. So, I think that went very well. If you have an open-minded team that collaborates well. Open minded to learning and listening to the family member and like advocating for the resident itself. (Canada, external facilitator)

if it’s for the benefit of the resident, they’ll drive it forward. I think the staff here are very eager to learn. (Republic of Ireland, director of nursing)

Staff who viewed the intervention as important were motivated to integrate the intervention into practice. Recognizing that the intervention was core to their work facilitated adoption into usual care:

Yeah ‘cos we’re hoping that we can we get the ok to continue it afterwards…I think it will benefit the home and ourselves as a whole if that’s on offer, all round. (UK—England, internal facilitator)

I think FCC (family care conference) will become routine because family carers provided positive feedback and expressed this desire. (Italy, internal facilitator)

The strength of the perceived value of the intervention facilitated the emergence of individuals in the nursing home who assumed the role of “champion” for the implementation. These individual(s) represented the “face” of the intervention, supporting, and driving the implementation effort. Staff engaged with the intervention and its implementation both formally and informally, identifying challenges and strategies to integrate the intervention into practice in the nursing home:

I think it was definitely helpful to have yourself as the you know, internal support on site at the facility. There was always that go to individual… we had a lot of face-to-face discussions and regular communications. So, to have that touch base with yourself was I think essential if not vital in all of its elements to support the success of the study. (Canada, internal facilitator)

### Theme 3. The Perceived Value of Building Staff Skills in Communication With Families

Promoting training among nursing home staff rested on making a clear distinction between how existing end-of-life care conversations transpired with family members and how training would improve their confidence and skills in the conversations they would have with family members:

I suppose the conversations we had with the family and it was about comfort care as opposed to let’s move away from this weighing every month and trying to get their weight up … yeah it was just you know let’s look at things differently, the family are on board here. (Republic of Ireland, internal facilitator)

Nursing home managers reported that accepting the intervention was dependent on the motivation of staff members to learn the skills to feel capable and comfortable engaging families in end-of-life discussions. This view promoted a sense of ownership and shared vision among nursing home staff on training and education in achieving change toward quality care in the nursing home:

I think it is very important, yes, that many, that people are given the tools on how to deal with it, and that they really have to be aware that it is extremely important for family members. (Netherlands, nursing home manager)

Anything that can help us continually improve because it is an area that’s been left in the dark a little bit I think. (UK—England, nursing home manager)

Family carers reported that good communication offered an understanding and eased stress and anxiety regarding their family member. Improving family–staff communications was not one-sided but benefited both family and staff who were better equipped to communicate end-of-life issues:

I have to really say that it helped me a lot, because it calmed me down a lot about my worries, like I was afraid my mom would suffer from pain, so she explained everything to me step by step. What are their options and also she told me that my mom already has some pain patches. (Czech Republic, family carer)

but one thing I would like to say is you know I would promote having this opportunity, I probably would not have raised the things that I have, I maybe would have just kept them in my own head, you know, this whole process has certainly supported me, in understanding you know what we can ask for. (UK—Northern Ireland, family carer)

### Theme 4. Factors External to the Nursing Home

The nursing homes’ reception and adoption of the intervention were influenced by conditions outside the nursing homes, for example, the infection control procedures that were deployed by nursing homes in response to the COVID-19 pandemic. COVID-19 had an impact on staff and family priorities, which sometimes meant that the intervention was a secondary consideration. Staff shortages and pressures on resources due to COVID-19 did undermine staff commitment to the project:

… at this moment, they [staff] are basically in survival mode. (Netherlands, staff)

Time is poor and nurses few. COVID has further worsened the situation because of extra calls for nurses from hospitals to manage the emergency. Thus, several nurses have transitioned into the hospital settings. (Italy, internal facilitator)

Due to the pandemic, training resources developed for nursing home staff included considerations to implement the intervention remotely (online or over the telephone). However, internal facilitators did not feel such an approach facilitated desired levels of discussion. Staff preferred to facilitate discussions in person in line with prevailing COVID-19 restrictions (social distancing, masks, testing), when possible. Similarly, family carers expressed the importance of holding these conversations with staff in person:

…one of the barriers especially during COVID is the fact that we complete these care conferences via online (…) we do not have the technology in place to do it on a regular basis. Plus we do not have sufficient IT support. (Canada, internal facilitator)

Yes, what I found a challenge was when we had to talk to those people via the laptop upstairs. (…) I thought I don’t know you at all and you just have to improvise in the middle of a conversation. (Netherlands, internal facilitator)

Nursing home staff and external facilitators reported the importance that the delivery of the training needed to be customized to suit conditions in the nursing home. This included being responsive to the unique learning needs of the nursing home staff, providing on-site training, whether face-to-face, digital, or hybrid as well as timing delivery of training and support to suit staff schedules:

My role as external felt critical to the implementation of the study. I helped develop tailor-made trainings, assisted with scheduling and communicating with family members, and supporting staff by role-modelling intervention and providing in-the-field support to help with KTE (Knowledge Translation and Exchange); build staff confidence and ease engaging in FCC (family care conference) discussions. (Canada, external facilitator)

### Theme 5. Resource Impact on Delivering the Intervention

An important consideration in facilitating uptake of the intervention was the nursing homes’ capacity to incorporate the required change in practice with the existing demands in the nursing home. Resource challenges in delivering the intervention were noted, with tasks taking longer than had been envisaged. Engaging and explaining the intervention to families, in particular, took more time than expected, and the process could be fragmented and extended because of needing to find time during in-person visits to speak with families, the potentially sensitive nature of the discussion, and the need to arrange case conference appointments around family availability and intermitted lockdowns due to COVID-19 outbreaks. Following up with families after case conferences, for example, with answers to queries, could also require more time than anticipated. Interviewees also talked about the time needed to prepare in advance for case conferences, which was sometimes undertaken in personal time:

I thought that my time would only be restricted to like my work hours, so the time I’m at work, but, yeah, so it took my time even outside work. Sorry, not just doing the training, so even before the care conference you have to prepare, so sometimes I just couldn’t do that at work, so I’d be [doing it at] home. (UK—Northern Ireland, internal facilitator)

In other cases, interviewees reported the intervention being delivered with paid overtime or through having paid staff covering usual duties:

[There] was extra cover needed on the unit just to cover it. (Republic of Ireland, director of nursing)

All meetings took place in extra-working hours [...], thereby it was not necessary to have more people. (Italy, internal facilitator)

Another challenge that staff described was of combining case conferences with day-to-day work on the floor, particularly where there were unexpected staff shortages:

The challenge is making sure that we have a registered staff member there like an RN (registered nurse) or an RPN (registered practical nurse) … Sometimes there wasn’t someone on shift, because we ran short. (Canada, internal facilitator)

It could be difficult to deliver the intervention where staff felt they might be interrupted or rushed. On occasion, to enable the case conference to proceed, managers or senior staff would need to step in to assist, which could impact on their other work. Staff made a number of observations concerning the sustainability of the intervention. Many emphasized the need for staff to have protected time, requiring explicit reallocation of their usual duties. Potentially, this could be, and was sometimes in practice, met through the use of paid overtime or use of cover staff. However, it was sometimes thought that this could be achieved through collaborative and flexible working between team members. It was also noted that having more staff trained and able to deliver the intervention increased the scope for such flexibility:

Time is a challenge everywhere but again, from experience, what we find is, by having proper channels of communication and I guess prioritising sharing information with families—whether that’s done informally or in a sit-down meeting—it actually ultimately, from a time point of view, saves you time in the end. (Republic of Ireland, internal facilitator)

Interviewees also saw scope for integrating the intervention into usual care, for example, annual review meetings with families. In some cases, the intervention was an extension of existing practices of regular discussions with families. These homes were more likely to have established practices that enabled them to effectively resource the case conferences:

If they put in as part of the ITM (interdisciplinary team meetings) is a suggestion that would be easier but if separate meeting, because they have so many different meetings, I am sure there would be a resistance (from staff) (…) and we cannot offer (1 hour meetings) to everyone. (Canada, internal facilitator)

I think yes they definitely want to continue it and they definitely feel it’s something that as a home they can offer. (UK—England, external facilitator)

I would definitely advocate that something like this is very beneficial for families who have loved ones who are approaching death. (Republic of Ireland, family caregiver)

### Economic Aspects of Implementation

Table 5 illustrates the cost of delivering the intervention at sites that participated in the study.

**Table 5.** Training: Facilitator Attendance, Hours, and Total and Unit Costs of Training Delivery (£, 2021)

Training Delivery Methods		
Items—costs, numbers of personnel attending, hours of attendance		Units
Training facilitators via the online training platform		
Costs		£
	External facilitator total costs	£3,472
	Internal facilitators total costs	£2,086
	All facilitators’ total costs	£5,558
Personnel		No./hours
	External facilitator total attenders	14
	External facilitator total attendance hours	93
	Internal facilitator total attenders	31
	Internal facilitator total attendance hours	96
	All facilitators total attenders	45
	All facilitators total attendance hours	189
Training internal facilitators face-to-face (online or in-person)		
Costs		£
	In-person training—total costs	£10,093
Personnel		No./hours
	Total in-person training attenders	30
	Total in-person training attendance hours	113
	Total in-person training attendances	73
Training external and internal facilitators across training delivery methods		
Costs		£
	Total costs of training excl. backfill	£15,651
	Total costs of training inc. backfill	£16,354
Personnel		No./hours
	Total number of internal facilitator attenders	46
	Total hours of internal facilitator attendances	209
	Mean hours of training per internal facilitator	5.4
Unit costs, online platform		£
	Costs per external facilitator	£248
	Costs per hour external facilitator training	£37
	Costs per internal facilitator	£67
	Costs per hour internal facilitator training	£22
	Costs per hour, any type of facilitator	£29
	Costs per any type of facilitator	£124
Unit costs, face to face		£
	Cost per internal facilitator	£336
	Cost per internal facilitator attendance	£138
	Cost per internal facilitator hour	£89

*Note*: excl = excluding; inc = including; No/hours = number of hours; £ = pound sterling.

Both external and internal facilitators accessed training tailored to their roles using the online training platform. External facilitators’ total hours on the platform (required to “train the trainers”) were similar to those of the internal facilitators, despite their fewer numbers. In addition, external facilitators provided internal facilitators with ongoing support either in-person, or online. Taking into account COVID-19 social distancing protocols training of internal facilitators was customized to optimize the available supportive conditions in the nursing homes. Usually in response to COVID-19 social distancing. Typically as a hybrid combining face-to-face and online. Most internal facilitators were trained using both face-to-face and online platform methods, receiving on average 5.4 hr of training each. Internal facilitators spent somewhat longer in face-to-face training than on the online platform (113 vs 96 hr). We did not collect data to assess satisfaction between the two modes of delivery. Costs of providing the face-to-face training sessions to internal facilitators were about five times those of using the training platform. The total costs of online platform and face-to-face training methods of the facilitators at the implementing sites were £15,651 (costs of all 46 internal facilitators, including nonimplementing sites, not shown in the table, were £16,904). Costs of training internal facilitators face-to-face were higher than those of the online platform training method, being £336 and £67 per facilitator, and £89 and £22 per facilitator hour, respectively. It should be noted, however, that the numbers and duration of face-to-face sessions varied considerably between countries, as did the number of hours of preparation time, venue, and materials costs, leading to a pronounced spread in these costs (the median total cost of training internal facilitators was £562, interquartile range £507). Total costs of training all facilitators, if including the costs of backfilling internal facilitators’ shifts, were slightly (4%) higher than if excluding these.

## Discussion and Implications

In this article, we identified the facilitators and barriers that support the implementation of the Family Carer Decision Support Intervention in nursing homes. This research study generated context-specific insights on taking a proven intervention and implementing it into nursing home practice. An outcome of this work was the development of a final set of theoretical propositions associated with five themes that were drawn from interviews provided by care home staff, family members, external facilitators, and healthcare professionals. The final propositions serve as implementation considerations for policymakers and nursing home practitioners.

The themes and final propositions that were identified in this work are supported in several models for improving program implementation (Berwick, 2003; Damschroder et al., 2009; Feldstein & Glasgow, 2008; Rycroft-Malone, 2004). Implementation research highlights how the perception of intervention characteristics influences the promotion of its use (Damschroder et al., 2009). In this study, nursing home staff identified the importance of evidence that the intervention benefited family carer decision-making. Adapting the intervention to suit the operating context was another supporting feature where nursing home staff recognized that the implementation process could be adapted to meet their workplace circumstances. Theme 3 documents the recognition by nursing home staff that the intervention addressed family carer priorities and that the intervention offered staff training opportunities to develop the necessary skills to communicate with families on goals of care for their family member.

The research literature has stressed the importance of facilitation in changing practice in nursing homes (Kinley et al., 2014). How facilitation may manifest itself is typically shaped by context. Facilitation describes (an) individual(s) internal or external to the nursing home who is dedicated to achieving success in the change effort and whose role is to identify and address organizational opportunities to support change or challenges such as staff inertia and resistance. Facilitation was instrumental in the implementation of the intervention and has been recommended as a key strategy for implementation of health innovations (Parmar et al., 2022). In this study, “high” facilitation was manifested with the use of trained facilitators external to the nursing homes who were available to support nursing home staff in training staff and the delivery of the intervention. It was not possible to assess the sustainability of the intervention in the nursing homes with the removal of external facilitation after the study period.

Organizational characteristics have been highlighted in the literature as a significant condition in the implementation process (Berwick, 2003). In our study, important organizational drivers included committed staff that were engaged in the implementation process and leadership that supported staff in the delivery of the intervention. A key organizational factor was the ability of the nursing home staff to accommodate the responsibilities of delivering the intervention within their regular workload. In some nursing homes, strategies to accommodate the implementation process included paid overtime, use of cover staff and promoting collaborative and flexible working between team members. These strategies were indicative of innovative leadership in the nursing home that facilitated implementation of the intervention supporting organizational change (Damschroder et al., 2009).

Cost is a central issue in service innovation and a concern among potential adopters of innovative practice (Feldstein & Glasgow, 2008). Understanding cost is challenging because it varies depending on the complexity of the intervention, the implementation strategies used, and the settings for delivery. In this study, we estimated the indicative costs of the train-the-trainer intervention. A comparison of face-to-face versus online training showed that online training offered a less expensive mode of delivering training.

Recipient response to innovation in practice has also been identified in the literature to maximize intervention effectiveness (Rycroft-Malone, 2004). In our study, family carers viewed the intervention as addressing a priority of improving the quality of communication with nursing home staff on care planning for their family member. Further, the importance of the quality of the relationship between nursing home staff and family carers was identified in this study.

Implementation research has reported that factors external to the organization can have a strong influence on the success of innovation in practice (Feldstein & Glasgow, 2008). The nursing homes’ reception and participation in the study were influenced by the COVID-19 pandemic. This manifested itself in infection control procedures deployed in the nursing homes that restricted engagement between staff, families, and researchers. The COVID-19 pandemic also had an impact on staff and family priorities, which meant that at times the intervention became a secondary consideration. Staff shortages and stress as well as COVID outbreaks placed pressures on the nursing homes that undermined commitment to the project.

This research engaged diverse stakeholders in order to examine the complex challenges they faced in the implementation of the intervention. The international nature of the study revealed the commonality of facilitators and barriers that influenced implementation of the intervention across several countries. Although the focus of this article has been on a cross-national analysis, we did note that the similarities in implementation issues were shared across countries. Detailed presentation on themes at the country level is beyond the scope of this article and will be pursued by country-level researchers in further work. The findings have benefits for decision-makers in this sector who are responsible for managing practice innovation and practitioners who ultimately have responsibility to implement the intervention, as well as researchers and educators who conduct research and teach implementation research.

A strength of this study was our effort to assess costs of both training and delivering the intervention through the use of timesheets for the internal and external facilitators. As part of the assessment of delivery costs we also collected time sheets maintained by the internal and external facilitators to assess levels of input required to deliver the intervention. Unfortunately, as a limitation, timesheets for the internal and external facilitators proved difficult to collect in a complete and consistent fashion across the participating sites thus preventing analyses.

Conducting this study during the COVID-19 pandemic had a significant impact on the implementation of the intervention. The COVID-19 pandemic created extraordinary conditions where initial planned implementation strategies were adjusted to develop social distancing protocols for staff training and how family care conferences were delivered. The use of digital technology, which was unplanned came to the fore and interactions between researchers and nursing home staff were at times constrained to “window visits.” Although characterized as a study challenge, the advantage of this experience was understanding practice innovation in a challenged practice environment.

There is consensus in implementation science that addressing contextual factors is critically important for understanding the translation of interventions into practice (Feldstein & Glasgow, 2008). However, there is little agreement on which contextual factors are key determinants of implementation outcomes. This study has identified the factors perceived by family carers, nursing home staff, and healthcare providers as the important drivers to consider when implementing the Family Carer Decision Support Intervention. A key learning from this study was also the recognition that nursing home context is dynamic and multiple factors influence implementation at different points of time.

## Data Availability

Qualitative data sharing is not possible due to ethical and confidential concerns. This study was not preregistered.
